# Ni-Based Hydrotalcite (HT)-Derived Cu Catalysts for Catalytic Conversion of Bioethanol to Butanol

**DOI:** 10.3390/ijms241914859

**Published:** 2023-10-03

**Authors:** Yan Xiao, Jie Li, Yuan Tan, Xingkun Chen, Fenghua Bai, Wenhao Luo, Yunjie Ding

**Affiliations:** 1 Hangzhou Institute of Advanced Studies, Zhejiang Normal University, 1108 Gengwen Road, Hangzhou 311231, China; xiaoyan@dicp.ac.cn (Y.X.); lijie_zjnu@163.com (J.L.);; 2 School of Chemistry and Chemical Engineering, Inner Mongolia University, 235 West University Street, Hohhot 010021, Chinaw.luo@imu.edu.cn (W.L.); 3 Dalian National Laboratory for Clean Energy, Dalian Institute of Chemical Physics, Chinese Academy of Sciences, Dalian 116023, China; 4 The State Key Laboratory of Catalysis, Dalian Institute of Chemical Physics, Chinese Academy of Sciences, Dalian 116023, China

**Keywords:** Ni-based hydrotalcite, biomass conversion, support effect, selectivity, stability

## Abstract

Catalytic conversion of biomass-derived ethanol into n-butanol through Guerbet coupling reaction has become one of the key reactions in biomass valorization, thus attracting significant attention recently. Herein, a series of supported Cu catalysts derived from Ni-based hydrotalcite (HT) were prepared and performed in the continuous catalytic conversion of ethanol into butanol. Among the prepared catalysts, Cu/NiAlO_x_ shows the best performance in terms of butanol selectivity and catalyst stability, with a sustained ethanol conversion of ~35% and butanol selectivity of 25% in a time-on-stream (TOS) of 110 h at 280 °C. While for the Cu/NiFeO_x_ and Cu/NiCoO_x_, obvious catalyst deactivation and/or low butanol selectivity were obtained. Extensive characterization studies of the fresh and spent catalysts, i.e., X-ray diffraction (XRD), Transmission electron microscopy (TEM), X-ray photoelectron spectroscopy (XPS) and Hydrogen temperature-programmed reduction (H_2_-TPR), reveal that the catalysts’ deactivation is mainly caused by the support deconstruction during catalysis, which is highly dependent on the reducibility. Additionally, an appropriate acid–base property is pivotal for enhancing the product selectivity, which is beneficial for the key process of aldol-condensation to produce butanol.

## 1. Introduction

Production of value-added fuels, chemicals and materials from abundant, renewable resources such as biomass and its derivatives could provide a feasible route to alleviate our dependence on dwindling fossil fuels [1,2]. Bioethanol is a promising platform molecule derived from biomass, which could be produced from the fermentation of sugar-containing crops [3,4]. Over the last decades, the catalytic upgrading of bioethanol into high-value-added chemicals, such as aldehyde, butanol, ethyl acetate and butadiene, has attracted great interest worldwide [5,6,7].

Butanol is an important commodity chemical which can be used for the production of a plethora of value-added end products with various applications [8,9]. It has been recognized as a promising alternative to gasoline owing to its excellent fuel properties and good compatibility [10,11]. Traditionally, butanol has been produced through oxo process (hydroformylation of propylene) or the fermentation of sugars—the acetone, butanol and ethanol (ABE) process [12,13]. However, the former depends heavily on the use of a fossil resource as raw material, and the latter process suffers from its long-time fermentation procedure and low production efficiency of butanol [12,13]. Alternatively, the catalytic upgrading of bioethanol into butanol through the Guerbet coupling process has attracted widespread attention both in academia and industry [14,15,16]. This is a well-known industrial route for higher alcohol synthesis and involves a tandem reaction route with several steps, including ethanol dehydrogenation to acetaldehyde, aldol condensation of acetaldehyde to 3-hydroxybutyraldehyde, dehydration of 3-hydroxybutyraldehyde to crotonaldehyde, and the hydrogenation of crotonaldehyde to butanol [17,18,19].

Many examples of heterogeneous catalysts are now available for the Guerbet coupling reaction, including hydroxyapatites [12,20], metal oxides [21,22], basic zeolites [23,24], and hydrotalcite (HT)-derived mixed oxides [25,26,27,28,29,30]. However, their catalytic performances are usually unsatisfactory due to the harsh reaction conditions [12,31,32]. The presence of transition metal active sites has been found to promote hydrogen transfer and greatly increase ethanol transformation [26,28,29]. Among these, Cu-based and Ni-based catalysts are promising candidates due to their low price and excellent hydrogenation capability [22,29,33]. Pang et al. have reported the effectiveness of Ni-MgAlO catalysts for continuous catalytic ethanol conversion to butanol [16]. The optimal Ni_4_MgAlO (19.5 wt.% Ni) catalyst shows 18.7% of ethanol conversion and 55.2% of n-butanol selectivity. G. W. Huber et al. investigated Cu/Mg_x_AlO_y_ catalysts for higher alcohol synthesis from ethanol, and found that the catalysts with low Cu loadings (0.1~0.6 wt.% Cu) show high selectivity to linear chain alcohols, while the catalyst with higher Cu loadings (>1.2 wt.% Cu) show high selectivity to ethyl acetate and acetone [27]. Recently, our group found that the addition of a small amount of Cu (>0.75 wt.%) to NiAlO_x_ can significantly improve the catalytic performance and afford a sustained ethanol conversion of ~35% and butanol selectivity of ~45% within 1000 h at 250 °C, 3 MPa N_2_ [33]. However, when the support of NiAlO_x_ was replaced by ZnAlO_x_, the product distribution changed greatly, with a large amount of ethyl acetate (Sel. of 42%) appearing in the system [34]. Previously, Boscolo et al. also found that the ethanol conversion, product selectivity and carbon deposition would be greatly affected by the support component, as the Mg^2+^ ion was partially substituted by Fe^2+^, Co^2+^, Ni^2+^, Cu^2+^ and Zn^2+^ ions [21]. These results strongly suggest that the role of support makes a large difference to catalytic performance. However, the origin of the support effect is still lacking evidence.

Herein, a series of Ni-based mixed-oxide-supported Cu catalysts were prepared and investigated for continuous catalytic conversion of ethanol into butanol. The activity, selectivity, and stability of different Cu/NiMO_x_ catalysts (i.e., Cu/NiAlO_x_, Cu/NiFeO_x_ and Cu/NiCoO_x_) were assessed and compared at 280 °C, 2 MPa N_2_. Notably, compared with Cu/NiFeO_x_ and Cu/NiCoO_x_, Cu/NiAlO_x_ outperforms markedly in butanol selectivity and stability. Based on systematic and complementary characterization studies of those fresh and spent catalysts, insights into the support effect on selectivity and stability were provided, together with the related reasons for the sustained superior performance of Cu/NiAlO_x_.

## 2. Results

### 2.1. N_2_ Physisorption Analysis of the Cu/NiMO_x_ Catalysts

**Figure 1:**
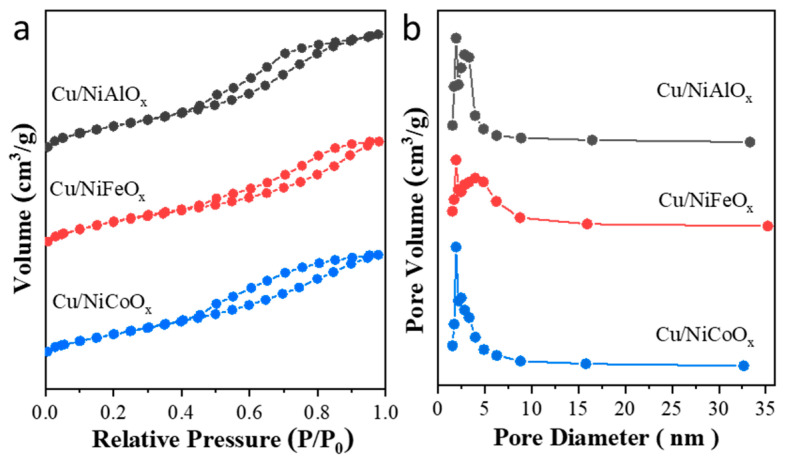
(**a**) N_2_ physical adsorption–desorption isotherms and (**b**) BJH pore size distributions of different supported copper catalysts.

The Ni-based HT-derived Cu catalysts (Cu/NiMO_x_), with a similar Cu loading of ~1.5 wt%, were synthesized via the hydrothermal deposition precipitation method. The textural properties of three catalysts (i.e., Cu/NiAlO_x_, Cu/NiFeO_x_ and Cu/NiCoO_x_) were characterized by N_2_ physical adsorption–desorption measurements. The results of specific surface area (S_BET_), pore volume and pore size are listed in Table 1. The S_BET_ of three catalysts falls in the range of 209 to 260 m^2^/g, as the Ni-based mixed oxides varied among Al, Fe and Co. Similar values of the pore volumes and pore sizes are also observed for all three of the catalysts, indicating a similar structure for all of the mixed oxides. Furthermore, the obvious type IV isotherms with H4 hysteresis loops observed for the three catalysts indicate the presence of mesopores in all catalysts (Figure 1a) [35]. This is in line with the appearance of mesopores with a pore width of 3 to 8 nm determined by the Barrett–Joyner–Halenda (BJH) method, shown in the pore size distribution curves (Figure 1b). The actual metal loadings of copper were determined by inductively coupled plasma atomic emission spectroscopy (ICP-AES), from which all catalysts show a similar value of ~1.5 wt.% (Table 1).

### 2.2. XRD Analysis of the Cu/NiMO_x_ Catalysts

**Figure 2:**
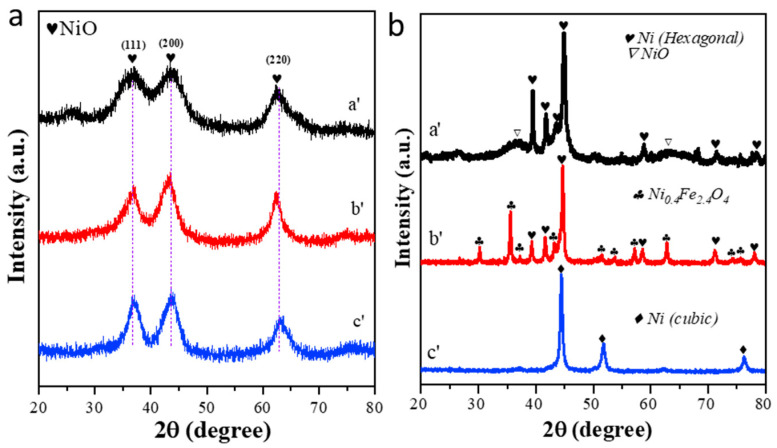
XRD patterns of different Ni-based HT-derived Cu catalysts before (**a**) and after (**b**) the reaction: (a’) Cu/NiAlO_x_; (b’) Cu/NiFeO_x_; (c’) Cu/NiCoO_x_.

The X-ray diffraction (XRD) patterns of Ni-based HT-derived Cu catalysts before and after the reaction are displayed in Figure 2. All catalysts display similar phase structures before the reaction, with diffraction peaks at 37.1°, 43.1° and 62.6°, respectively, which are the characteristic reflections of the (111), (200) and (220) crystal phases of NiO (Figure 2a). Furthermore, before the reaction, no diffraction peaks of metallic Cu can be observed, implying that the homogeneous distribution of Cu species or the Cu content is below the detection limit. For the spent Cu/NiAlO_x_, Cu/NiFeO_x_ and Cu/NiCoO_x_ catalysts, it can be found that the metallic Ni clearly appeared. The diffraction peaks at 39.7°, 41.8° and 45.1° can be assigned to the characteristic reflections of (100), (002) and (101) planes of hexagonal Ni. The diffraction peaks at 44.5°, 51.8° and 76.3° can be due to the reflections of (111), (200) and (220) planes of cubic Ni (Figure 2b). For the spent Cu/NiAlO_x_ and Cu/NiFeO_x_ catalysts, both display mixed phases of hexagonal Ni and oxides. However, for the spent Cu/NiCoO_x_, only bulk Ni can be detected, evidenced by the symmetrical and sharp diffraction peaks of cubic Ni. As significantly reduced Ni phases appeared in all catalysts, the reduction atmosphere during the Guerbet coupling process can be confirmed. This is consistent with previous studies in which the H-transfer process appeared in the Guerbet coupling process, thereby producing in-situ formed hydrogen (from ethanol dehydrogenation) and playing a role as reducing agent [36]. Thus, the nickel species can be reduced during catalysis. However, the metallic Cu cannot be observed, which might be attributed to the low content. Furthermore, according to the Scherrer equation, the larger Ni particle sizes of the spent Cu/NiFeO_x_ and Cu/NiCoO_x_ (~30 nm) catalysts can be determined compared with those of the spent Cu/NiAlO_x_ (~20 nm), implying a severe agglomeration of Ni species for the Cu/NiFeO_x_ and Cu/NiCoO_x_ during catalysis.

### 2.3. TEM Analysis of the Cu/NiMO_x_ Catalysts

**Figure 3:**
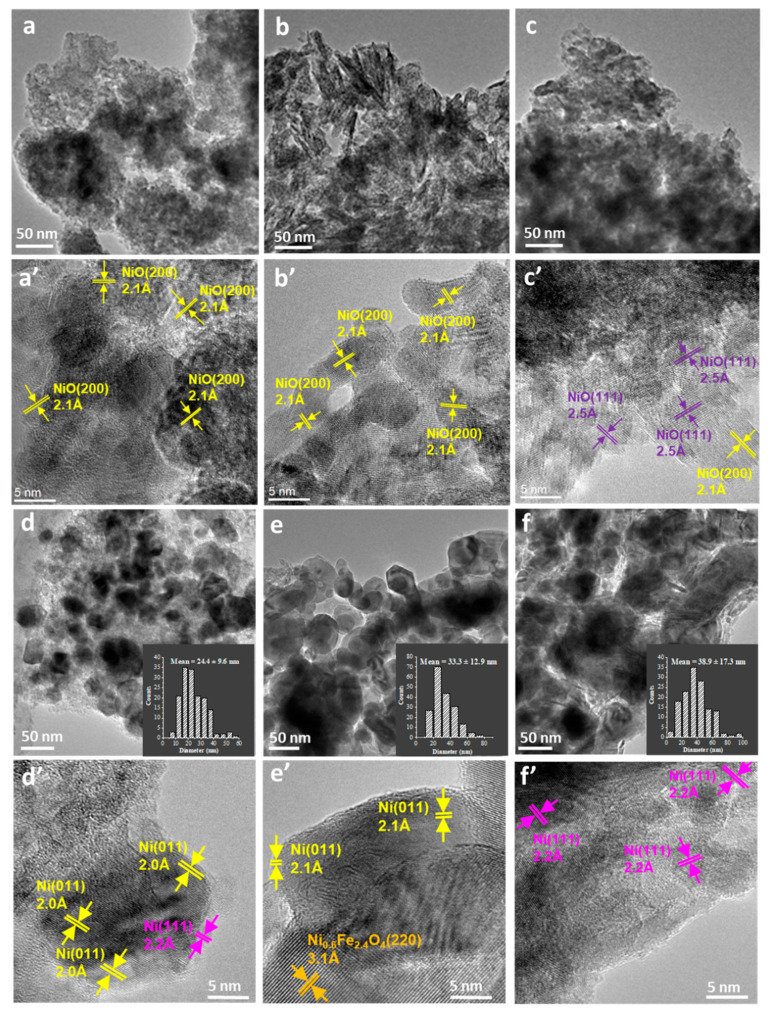
TEM (**a**–**f**) and corresponding HRTEM (**a’**–**f’**) images of different supported copper catalysts before (**a**–**c**) and after (**d**–**f**) the reaction. (**a**,**d**) Cu/NiAlO_x_; (**b**,**e**) Cu/NiFeO_x_; (**c**,**f**) Cu/NiCoO_x_.

Transmission electron microscopy (TEM) and high-resolution TEM (HRTEM) were employed to study the microstructures of the Cu/NiMO_x_ catalysts before and after the reaction. In the fresh catalysts, no big metal particles could be observed in TEM images, as displayed in Figure 3a–c. By calculating the lattice parameter of the HRTEM images (Figure 3a’–c’), NiO(200) and NiO(111), with interplanar crystal spacings of ~2.1 Å and ~2.5 Å, respectively, could be discerned in all catalysts. This indicates that Ni existed as NiO and was homogeneously dispersed on the catalysts, which in turn agrees well with the XRD results (Figure 2a). After the reaction, Ni particles appeared in all spent catalysts (Figure 3d–f), with the interplanar crystal spacing of ~2.0 Å for Ni(011) and ~2.2 Å for Ni(111) (Figure 3d’–f’). This indicates that a severe support reduction occurs in the Cu/NiMO_x_ catalysts. The average Ni particle sizes of the spent Cu/NiAlO_x_ (24.4 nm, Figure 3d) are much smaller than those of the spent Cu/NiFeO_x_ (33.3 nm, Figure 3e) and Cu/NiCoO_x_ (38.9 nm, Figure 3f), which is consistent with the XRD results (Figure 2b) and implies that the Ni agglomeration is more significant over the Cu/NiFeO_x_ and Cu/NiCoO_x_ than that of the Cu/NiAlO_x_ during catalysis.

### 2.4. XPS Analysis of the Cu/NiMO_x_ Catalysts

**Figure 4:**
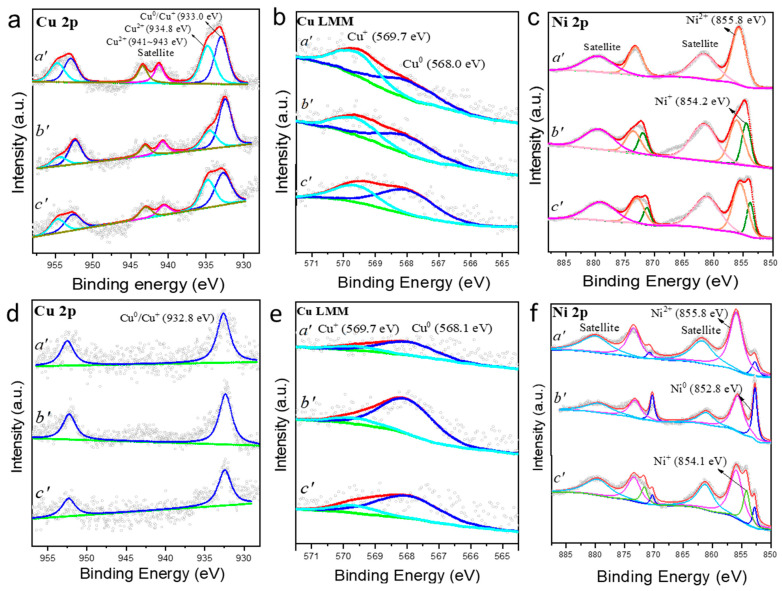
(**a**,**d**) Cu 2p XPS, (**b**,**e**) Cu LMM and (**c**,**f**) Ni 2p XPS of different Cu/NiMO_x_ catalysts before (**a**–**c**) and after (**d**–**f**) catalytic tests: (a’) Cu/NiAlO_x_; (b’) Cu/NiFeO_x_; (c’) Cu/NiCoO_x_.

The chemical states of Cu and Ni in the fresh and spent Cu/NiMO_x_ catalysts were examined by X-ray photoelectron spectroscopy (XPS). The results are displayed in Figure 4. From Cu 2p XPS of the fresh Cu/NiAlO_x_, Cu/NiFeO_x_ and Cu/NiCoO_x_ catalysts, the characteristic peaks assigned to the Cu^2+^ (Cu 2p_3/2_) ions appear at 934.8 eV, with satellite peaks at 941~943 eV [37,38]. The other peak, positioned at 933.0 eV, was assigned to the Cu^+^/Cu^0^ species [37,38]. Before reaction, it can be found that the Cu^2+^ and Cu^+^/Cu^0^ species coexisted on the catalysts (Figure 4a). However, after reaction, most of the Cu^2+^ species were reduced to Cu^+^/Cu^0^ species, with the absence of satellite peaks of Cu^2+^ (Figure 4d).

Since the binding energies of Cu 2p for the Cu^+^ and Cu^0^ are very close to each other, the Auger electron spectroscopy (AES) peaks of Cu LMM were measured to distinguish Cu^+^ from Cu^0^. The peaks positioned at 568.0 eV and 569.7 eV featured the peaks of Cu^0^ and Cu^+^, respectively [39]. Before reaction, Cu^0^ and Cu^+^ species coexisted on all catalysts (Figure 4b). The ratios of Cu^0^/Cu^+^ over the Cu/NiAlO_x_, Cu/NiFeO_x_ and Cu/NiCoO_x_ are 0.93, 1.34 and 1.81, respectively. After reaction, most of the copper oxides are reduced (Figure 4e), with the ratios of Cu^0^/Cu^+^ increasing up to 4.18, 7.33 and 4.76. The results suggest that Cu species can also be reduced during catalysis. Additionally, Cu^0^ is predominant specie over the surface of the spent catalysts.

Additionally, in terms of Ni, two strong peaks at 873.3 and 855.8 eV appeared, accompanying two shakeup satellites at 879.7 and 861.8 eV (Figure 4c) and demonstrating the presence of Ni^2+^ [40,41]. Furthermore, the bands at 854.2 eV and 852.8 eV can be assigned to Ni^+^ and Ni^0^, respectively [41,42]. From Figure 4f, the reduction peak of Ni^2+^ to Ni^0^ can be observed in all Cu/NiMO_x_ catalysts, indicating the severe support reduction of Cu/NiMO_x_ upon catalysis. This agrees well with the XRD and HRTEM results. However, for the Cu/NiFeO_x_ and Cu/NiCoO_x_, Ni^+^ can be observed in the fresh catalysts (Figure 4c), and the amounts of Ni^0^ species are clearly increased with the spent Cu/NiFeO_x_ and Cu/NiCoO_x_ in comparison with those of the Cu/NiAlO_x_. This indicates that Cu/NiFeO_x_ and Cu/NiCoO_x_ possess much stronger reducibility, which might cause the agglomeration of metal particles and the severe destruction of support, in turn leading to poor stability of the catalysts [43].

### 2.5. H_2_-TPR Results of the Cu/NiMO_x_ Catalysts

**Figure 5:**
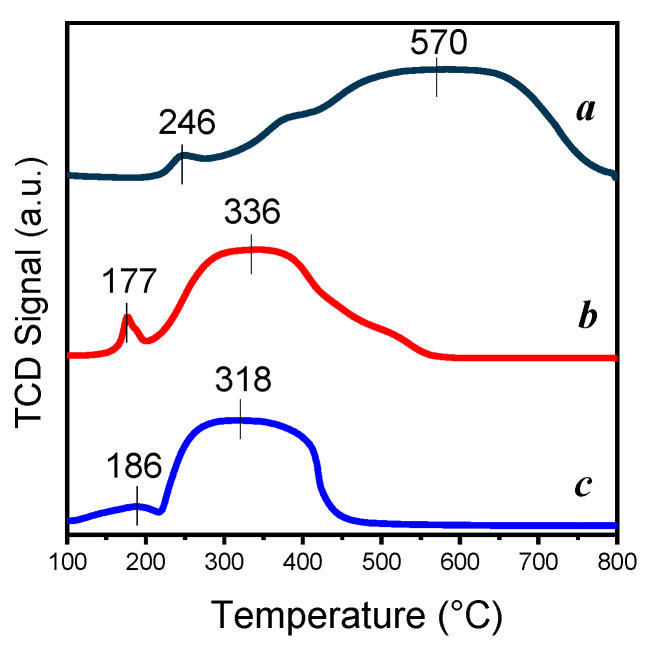
H_2_-TPR profiles of different supported copper catalysts: (a) Cu/NiAlO_x_; (b) Cu/NiFeO_x_; (c) Cu/NiCoO_x_.

Hydrogen temperature programmed reduction (H_2_-TPR) was conducted to measure the reducibility of Cu/NiMO_x_ catalysts. As shown in Figure 5, two major consumption peaks were observed in all catalysts. The peaks at 150~246 °C can be attributed to the reduction of copper oxides [33,38]. This suggests that the Cu species is easily reduced under the reaction conditions (280 °C, 2 MPa). However, the reduction temperature of copper oxides over the Cu/NiAlO_x_ shifted from 246 °C to 177 °C (Cu/NiFeO_x_) and 186 °C (Cu/NiCoO_x_,), indicating that Cu is easier to reduce than the latter two catalysts. Furthermore, wide and broad peaks are attributed to support reduction shifts from 450–750 °C for Cu/NiAlO_x_ to 200–500 °C for Cu/NiFeO_x_ and Cu/NiCoO_x_ [44,45,46], pointing to the facile reducibility of Cu/NiFeO_x_ and Cu/NiCoO_x_ under H_2_ atmosphere and agreeing well with the XPS results.

### 2.6. CO_2_/NH_3_-TPD Results

**Figure 6:**
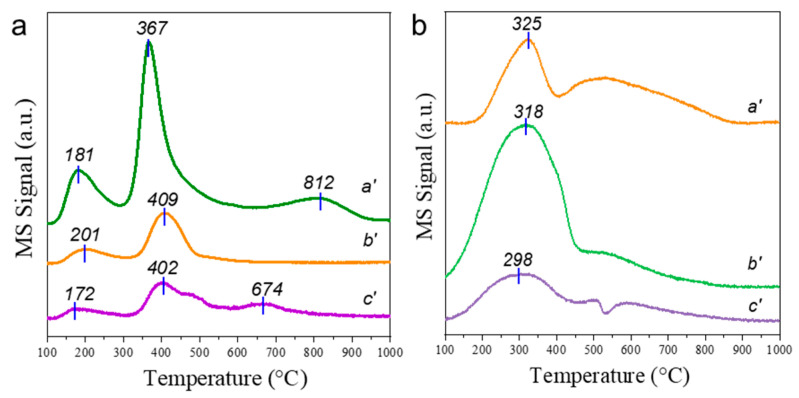
(**a**) CO_2_-TPD and (**b**) NH_3_-TPD profiles of different Ni-based supports: (a’) NiAlO_x_; (b’) NiFeOx; (c’) NiCoO_x_.

The acid/base sites are reported to play an essential role in modulating the catalytic performance, especially when tailoring the selectivity of the Guerbet coupling process [47,48]. As the Cu content is similarly low (~1.5 wt.%) in all catalysts, the acid/base property is largely derived from the corresponding supports. Thus, to evaluate the acid/base properties, CO_2_-TPD and NH_3_-TPD measurements were conducted for the different Ni-based oxides. Figure 6a depicts the CO_2_-TPD profiles of the above NiMO_x_ supports. Three types of basic sites (i.e., weak basic site: <300 °C, medium basic site: 300~500 °C and strong basic site: >500 °C) can be observed in the profiles [41,49]. The specific amounts of each basic site were calculated by integrating the corresponding peak areas and are summarized in Table 2. Amongst these, the NiAlO_x_ possesses the largest amount of basic sites, with weak basic sites of 76.7 μmol/g, medium basic sites of 280.9 μmol/g and strong basic sites of 56.2 μmol/g. Whereas for the NiFeO_x_ and NiCoO_x_, a clear decrease in the amounts of basic sites can be observed, with total basicity of 110~130 μmol/g.

Beyond the basic sites, the amount and strength of the acid sites are also essential for tailoring the products distributions [27,50]. Figure 6b shows the NH_3_-TPD profiles of the three NiMO_x_ supports. From these results, all of the samples show roughly two types of acid sites (i.e., weak acid sites: <450 °C and strong acid sites: >450 °C) [41]. The number of each acid site over the different NiMO_x_ are summarized in Table 2. For the NiFeO_x_, the largest amount of weak acidic sites (131.6 μmol/g) is shown when compared with those of NiAlO_x_ (42.0 μmol/g) and NiCoO_x_ (37.3 μmol/g). This might decrease the selectivity of the Guerbet coupling process, as the acid sites are often reported to be beneficial for the dehydration reaction in producing ether and ethyl acetate [19,34].

### 2.7. Catalytic Performance of Different Catalysts in Ethanol Coupling to Butanol

**Figure 7:**
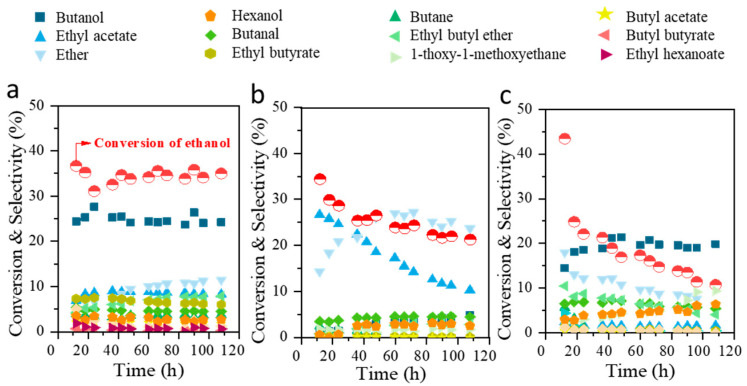
Catalytic performances of different supported Cu catalysts for ethanol coupling to butanol: (**a**) Cu/NiAlO_x_; (**b**) Cu/NiFeO_x_; (**c**) Cu/NiCoO_x_. Reaction conditions: catalyst: 1 g, pressure of N_2_: 2 MPa, reaction temperature: 280 °C, GHSV: 692 h^−1^, LHSV: 4.8 h^−1^.

The catalytic performances of ethanol coupling to butanol over the Cu/NiMO_x_ catalysts were accessed in a continuous fixed-bed reactor at 280 °C, 2 MPa of N_2_. Among the evaluated catalysts, Cu/NiAlO_x_ displays the best performance in terms of activity, selectivity and stability, with a sustained ethanol conversion of ~35%, butanol productivity (BP) of ∼719.3 mmol·g_cat_^−1^·h^−1^, and butanol selectivity of 25% in a time-on-stream (TOS) of 110 h (Figure 7a). Ether, ethyl acetate, ethyl butyl ether, ethyl butyrate, butanal, etc. are detected as the by-products. For the Cu/NiFeO_x_, a progressive drop-in activity from an initial ethanol conversion of 34% to 10% and a lower BP of 139.7 mmol·g_cat_^−1^·h^−1^ are observed upon a TOS of 110 h (Figure 7b). Considerable amounts of ethyl acetate and ether, rather than butanol, are obtained as the main products after catalysis, indicating a poor performance of Cu/NiFeO_x_ in terms of butanol selectivity (<5%). For the Cu/NiCoO_x_, a high initial ethanol conversion of 43.4% and a quick decrease in activity from an initial ethanol conversion of 43.4% to 25% (at a TOS of 12 h) and further to 10% (at a TOS of 110 h) are observed during catalysis (Figure 7c). A sustained butanol selectivity in the range of 15% to 21%, is observed for the reaction, with a BP of 535.2 mmol·g_cat_^−1^·h^−1^ over the Cu/NiCoO_x_. Although a sustained butanol selectivity of 15~21% can be observed for Cu/NiCoO_x_, a gradual deactivation of Cu/NiCoO_x_, in particular with respect to its activity, is illustrated by the long-term 110 h continuous run. It is clear, therefore, that the catalytic performances of the Cu/NiMO_x_ catalysts for the Guerbet coupling reaction were affected by the support effect. Cu/NiAlO_x_ outperforms in catalytic performance in terms of activity, selectivity and stability, when compared with Cu/NiFeO_x_ and Cu/NiCoO_x_.

## 3. Discussion

According to our previous studies, the support itself displays poor catalytic activity for ethanol upgrading to higher alcohols [33,34]. The introduction of an appropriate amount of Cu species (>0.75 wt.%) can significantly increase the active sites for ethanol dehydrogenation and crotonaldehyde hydrogenation, thereby enhancing the ethanol conversion and the production of the target product [33]. In this work, the addition of ~1.5 wt.% Cu is adequate for ethanol transformation, as the initial ethanol conversion over the three Cu/NiMO_x_ catalysts did not show many differences (Figure 7). However, after a TOS of 110 h, clear catalyst deactivation appeared over the Cu/NiFeO_x_ and Cu/NiCoO_x_ but not the Cu/NiAlO_x_, indicating that the support effect greatly affected the stability of the catalysts. Based on our characterization results, all catalysts show different degrees of reduction under the reaction conditions, as proved by XRD (Figure 2), TEM (Figure 3) and XPS (Figure 4). However, over the Cu/NiFeO_x_ and Cu/NiCoO_x_, a stronger and clear reducibility was observed, as proved by XPS (Figure 4) and H_2_-TPR (Figure 5). This might cause the severe support deconstruction under H_2_ atmosphere and induce the agglomeration of Cu/Ni particles, leading to poor stability of the corresponding catalysts.

**Scheme 1:**
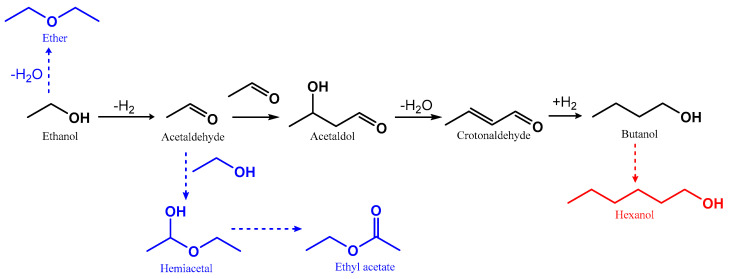
The reaction path of the Guerbet coupling process over the Cu/NiMO_x_ catalysts.

Furthermore, in terms of butanol selectivity, the product distribution was strongly dependent on the support selection. Over the Cu/NiAlO_x_, a decent butanol selectivity can be obtained (Figure 7a), whereas over the Cu/NiFeO_x_, ethyl acetate and ether were produced as the main products (Figure 7b). Based on the previous reports, the product distribution of the Guerbet coupling process was highly correlated with the acid–base properties of the catalysts [51,52,53]. Additionally, their catalytic performances are boosted by an appropriate acid–base cooperation [40,52]. In our work, the CO_2_/NH_3_-TPD results indicate that different Ni-based supports show quite different amounts and strengths among the acid/base sites (Figure 6). Among these, the NiAlO_x_-supported Cu catalyst possesses the optimal acid and basic sites, with a majority of them either of weak or medium strength. This is proposed to be of benefit when catalyzing aldol condensation and improving the butanol selectivity [51,53]. Cu/NiCoO_x_ is inferior to Cu/NiAlO_x_, showing a lower butanol selectivity of between 15~21% during a TOS of 110 h. It can be deduced that the decreased basic and acid sites lead to a reduction of C-C coupling production, thus decreasing the butanol selectivity. However, for the Cu/NiFeO_x_, the presence of excessive acid sites leads to a severe dehydration process, which greatly improves the generation of dehydration product, such as ethyl acetate and ether [54]. The proposed reaction mechanism of the Cu/NiMO_x_ catalysts for the Guerbet coupling process is presented in Scheme 1, from which we can see that multifunctional sites are required for the synergistic catalysis of the Guerbet coupling process. The Cu species provide active sites for ethanol dehydrogenation and crotonaldehyde hydrogenation, while supports with appropriate acid and/or basic properties supply active sites for the promotion of aldol condensation to produce butanol.

**Figure 8:**
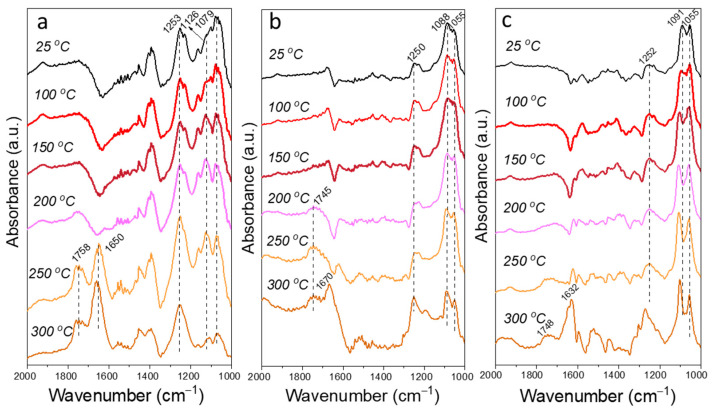
In situ DRIFTS of ethanol over different supported Cu catalysts in the range of 1000~2000 cm^−1^: (**a**) Cu/NiAlO_x_; (**b**) Cu/NiFeO_x_; (**c**) Cu/NiCoO_x_.

To dynamically track the intermediates during the ethanol coupling process, in situ DRIFTS measurements using ethanol as the probe molecule were conducted for different Cu/NiMO_x_ catalysts (Figure 8). The spectra were obtained by subtracting the background obtained at room temperature and atmospheric pressure. After introducing ethanol with a He flow at 25 °C, the IR cell temperature was gradually elevated from 25 °C to 300 °C at a heating ramp of 10 °C/min. Simultaneously, the spectra were collected at respective steady states of 100, 150, 200, 250, and 300 °C. Notably, two featured bands at around 1055 and 1126 cm^−1^, corresponding to the C-O stretching vibrations of adsorbed ethoxide [29,51]; a band at around 1253 cm^−1^ that can be attributed to the δ(C-OH) of adsorbed ethanol/3-hydroxybutanal/butanol [26]; and two bands at around 1632 and 1758 cm^−1^ attributed to C=C and C=O groups of adsorbed crotonaldehyde [29,55]. For the Cu/NiAlO_x_ (Figure 8a), as the temperature increased from 25 to 300 °C, the bands at around 1126 cm^−1^ and 1079 cm^−1^ greatly decayed, while the band at 1650 cm^−1^ and 1758 cm^−1^ appeared and developed, indicating the consumption of adsorbed ethoxide and the production of intermediates such as 3-hydroxybutanal and crotonaldehyde. Nevertheless, minor changes in C-O, C=C and C=O vibrations are observed for Cu/NiFeO_x_ (Figure 8b) and Cu/NiCoO_x_ (Figure 8c) even as the temperature increased up to 250 °C. This implies a lower reactivity and butanol selectivity for Cu/NiFeO_x_ and Cu/NiCoO_x_ than of Cu/NiAlO_x_, which agrees well with the catalytic performances.

## 4. Materials and Methods

### 4.1. Materials

Copper nitrate trihydrate, Nickel nitrate hexahydrate, Ferric nitrate nonahydrate, Cobalt nitrate hexahydrate, butanol, ethanol and methanol were purchased from Sinopharm company (Beijing, China). Sodium hydroxide, sodium carbonate, aluminum nitrate hydrate, ethyl acetate and ortho-xylene were purchased from Aladdin Industrial Corporation (Shanghai, China). All chemicals were utilized without purification process.

### 4.2. Preparation of the Catalysts

#### 4.2.1. Synthesis of Different Ni-Based HTs

Different Ni-based HTs were prepared via the co-precipitation method. Amounts of 0.105 mol of Ni(NO_3_)_2_·6H_2_O and 0.035 mol of M(NO_3_)_3_·xH_2_O (M = Al, Fe, Co) were mixed with 150 mL of ultrapure water to obtain solution A. Amounts of 0.057 mol of Na_2_CO_3_ and 0.219 mol of NaOH were mixed with 150 mL of ultrapure water to obtain solution B. In a water bath at 80 °C, solution A was dropwise added into solution B with a rate of 50 mL/min using a constant flow pump. Then, the gel was stirred and aged at 80 °C for 24 h. The resulting suspension was filtered and exhaustively washed with deionized water until the pH of the filtrate reached 7. Then the filter cake was dried in an oven at 80 °C for 8 h to obtain NiM-HT (M = Al, Fe, Co).

#### 4.2.2. Synthesis of Ni-Based HT-Derived Cu Catalysts

The different supported Cu catalysts were synthesized through deposition precipitation method. Firstly, 2 g of the NiM-HT powders and 100 mL of deionized water were added into a round-bottom flask. Then, the pH was adjusted to ~10 by solution of Na_2_CO_3_. Subsequently, 80 mg of Cu(NO_3_)_2_·3H_2_O was added into the flask and the solution was heated to 80 °C. The reaction was processed for 2 h under constant stirring. Then, the product was filtered and washed with large amounts of water. Before tests, the samples were dried at 80 °C for 8 h and calcined at 300 °C for 2 h. The obtained samples were denoted as Cu/NiMO_x_ (M = Al, Fe, Co).

### 4.3. Catalytic Evaluation

The catalytic transformation of ethanol to butanol was carried out in a fixed-bed reactor with an inner diameter of 10 mm and a length of 660 mm. The reaction process was performed at specified conditions with N_2_ as the carrier gas. For each test, 1 g of the catalyst was loaded into the middle of the reactor. Both ends were filled with quartz sand. Before the test, N_2_ was introduced into the system and the pressure was maintained with a back-pressure regulator. Then, the temperature was programmed to increase to a specific value, with a heating ramp of 5 °C/min. Ethanol was then introduced into the system with constant liquid hourly space velocity (LHSV) by using a plunger pump (NP-KX-210). The analysis of products was conducted at an interval of 12 h. An Agilent 7890B chromatograph and mass spectrometer (Agilent 5977B MSD) were used offline to detect the reactants and products and were equipped with a flame ionization detector (FID) and an HP-5 capillary column (30 m × 0.32 mm). O-xylene was used as the internal standard.

### 4.4. Characterization

The actual Cu loadings were measured with an inductively coupled plasma atomic emission spectroscopy (ICP-AES) on an IRIS Intrepid II XSP instrument (Thermo Electron Corporation, Waltham, MA, USA). A Quantachrome NT3LX-2 instrument (FL, USA) was utilized to determine the textural properties of the catalysts by N_2_ physical adsorption–desorption at −196 °C. The phase structure and crystallinity of the fresh/spent catalysts were analyzed using X-ray diffraction (XRD). The spectra were recorded on a PW3040/60 X’Pert PRO (PANalytical, Almelo, The Netherlands) diffractometer with a Cu Kα X-ray source radiation at 40 kV and 30 mA in the range of 10°~90°. Transmission electron microscopy (TEM) and high-resolution transmission electron microscopy (HRTEM) were recorded on a JEOL JEM-2100F microscope (Tokyo, Japan) and Oxford detectors at 200 kV, respectively. The X-ray photoelectron spectroscopy (XPS) and X-ray-induced Auger electron spectroscopy (XAES) were conducted on an ESCLALAB 250Xi X-ray photoelectron spectrometer (Thermo Fisher Scientific, Madison, WI, USA) connected to a monochromatic Al and double anode Al/Mg ray source. The C 1s at 284.6 eV was recorded to adjust the binding energies of the other investigated elements. Hydrogen temperature-programmed reduction (H_2_-TPR) experiments were studied on a Micromeritics Autochem II 2920 chemisorber (Norcross, GA, USA). For each test, the catalyst (~100 mg) was treated at 300 °C for 1 h in a quartz U-tube reactor with a He gas stream (30 mL/min). After cooling to room temperature, a 10% H_2_/Ar flow (30 mL/min) was introduced into the system, and the sample was heated to 800 °C at a ramping rate of 10 °C/min. The H_2_-TPR signal was recorded by TCD and MS detector simultaneously. The basicity and acidity of catalysts were measured by temperature-programmed desorption (TPD) of CO_2_/NH_3_ on a Micromeritics Autochem II 2920 (Norcross, GA, USA) equipped with a thermal conductivity detector (TCD) and MS detector. Prior to the measurements, ~100 mg of catalyst was treated at 300 °C for 1 h in He (50 mL/min). Then, the temperature was cooled to 100 °C and the sample was adsorbed and saturated with 10% CO_2_/He or NH_3_/He flow pulse (30 mL/min) for 20 times. Subsequently, the sample was purged with He (30 mL/min) for 30 min, and a CO_2_/NH_3_-TPD signal was recorded from 100 to 1000 °C, with a heating rate of 10 °C/min with a TCD and an MS detector. In situ diffuse reflectance infrared Fourier-transformed spectroscopy (DRIFTS) was performed on a Bruker INVENIO Fourier-transform infrared spectrometer (Bruker, Karlsruhe, Germany), equipped with an MCT detector. Before the experiment, the catalyst was treated at 300 °C for 0.5 h. After cooling to room temperature, the background spectrum was recorded, and ethanol was introduced into the cell with the assistance of a He flow (30 mL/min). Then, the reaction temperature was increased gradually with a heating rate of 10 °C/min. After achieving a fixed temperature, the spectra were collected at respective steady states of 100, 150, 200, 250, and 300 °C.

## 5. Conclusions

In summary, we have prepared a series of supported Cu catalysts derived from Ni-based hydrotalcite (Cu/NiAlO_x_, Cu/NiFeO_x_ and Cu/NiCoO_x_) via the hydrothermal precipitation method. The catalytic performances for ethanol coupling to butanol were evaluated and compared with three different catalysts at the continuous fixed-bed reactor. The support effect was found to greatly influence the catalytic performance in terms of selectivity and stability. Notably, Cu/NiAlO_x_ outperforms in the butanol yield and long-term stability, when compared with Cu/NiFeO_x_ and Cu/NiCoO_x_. Furthermore, we have demonstrated that the appropriate acid–base combinations in close proximity within the support are pivotal for achieving a decent butanol selectivity in the Guerbet coupling process, while the deconstruction of the support by reduction during catalysis is the main reason for the deactivation of catalysts. Our findings may be of great help for the rational design of efficient heterogeneous catalysts with combination of multiple functional sites that can afford improved performance, especially in terms of selectivity and stability, in the tandem conversion of biomass-derived small substrates, including ethanol and beyond.

## Figures and Tables

**Table 1:** Textural properties of different supported copper catalysts.

Entry	Catalysts	Cu Loadings (%) ^a^	Specific Surface Area (m^2^/g) ^b^	Total Volume (cm^3^/g) ^b^	Pore Size (nm) ^b^
1	Cu/NiAlO_x_	1.6	260	0.29	1.93
2	Cu/NiFeO_x_	1.5	223	0.23	1.92
3	Cu/NiCoO_x_	1.3	209	0.24	1.93

^a^ As determined by ICP-AES; ^b^ As determined by N_2_ physisorption.

**Table 2:** The acid/base properties of different Ni-based supports.

Entry	Samples	Basic Sites (μmol/g) ^a^			Acidic Sites (μmol/g) ^b^	
		Weak Basic Sites	Medium Basic Sites	Strong Basic Sites	Weak Acidic Sites	Strong Acidic Sites
1	NiAlO_x_	76.7	280.9	56.2	42.0	53.2
2	NiFeO_x_	22.6	88.0	0	131.6	23.5
3	NiCoO_x_	29.6	79.7	20.3	37.3	18.6

^a^ As determined by CO_2_-TPD; ^b^ As determined by NH_3_-TPD.

## Data Availability

Data are available upon request from the corresponding authors.
